# Signatures within the esophageal microbiome are associated with host genetics, age, and disease

**DOI:** 10.1186/s40168-018-0611-4

**Published:** 2018-12-17

**Authors:** Nandan P. Deshpande, Stephen M. Riordan, Natalia Castaño-Rodríguez, Marc R. Wilkins, Nadeem O. Kaakoush

**Affiliations:** 1 Systems Biology Initiative, School of Biotechnology and Biomolecular Sciences, UNSW Sydney, Sydney, NSW 2052 Australia; 2 Gastrointestinal and Liver Unit, The Prince of Wales Hospital, Randwick, NSW 2031 Australia; 3 School of Biotechnology and Biomolecular Sciences, UNSW Sydney, Sydney, NSW 2052 Australia; 4 Ramaciotti Centre for Gene Function Analysis, UNSW Sydney, Sydney, NSW 2052 Australia; 5 School of Medical Sciences, UNSW Sydney, Kensington, Sydney, NSW 2052 Australia

**Keywords:** Esophagus, Microbiota, Community types, Metagenomics, Single nucleotide polymorphisms

## Abstract

**Background:**

The esophageal microbiome has been proposed to be involved in a range of diseases including the esophageal adenocarcinoma cascade; however, little is currently known about its function and relationship to the host. Here, the esophageal microbiomes of 106 prospectively recruited patients were assessed using 16S rRNA and 18S rRNA amplicon sequencing as well as shotgun sequencing, and associations with age, gender, proton pump inhibitor use, host genetics, and disease were tested.

**Results:**

The esophageal microbiome was found to cluster into functionally distinct community types (esotypes) defined by the relative abundances of *Streptococcus* and *Prevotella*. While age was found to be a significant factor driving microbiome composition, bacterial signatures and functions such as enrichment with Gram-negative oral-associated bacteria and microbial lactic acid production were associated with the early stages of the esophageal adenocarcinoma cascade. Non-bacterial microbes such as archaea, *Candida* spp., and bacteriophages were also identified in low abundance in the esophageal microbiome. Specific host SNPs in *NOTCH2*, *STEAP2-AS1*, and *NREP* were associated with the composition of the esophageal microbiome in our cohort.

**Conclusions:**

This study provides the most comprehensive assessment of the esophageal microbiome to date and identifies novel signatures and host markers that can be investigated further in the context of esophageal adenocarcinoma development.

**Electronic supplementary material:**

The online version of this article (10.1186/s40168-018-0611-4) contains supplementary material, which is available to authorized users.

## Background

Esophageal adenocarcinoma (EAC) is one of the two main histological types of esophageal cancer. The incidence of EAC has substantially increased since the 1970s [1], and this rise represents a real increase in disease burden unrelated to over-diagnosis or reclassification [2].

EAC is the final stage of a cascade of events that begin with gastroesophageal reflux disease (GERD), a condition where the esophagus is chronically exposed to acid, bile, and other stomach contents [3]. This results in inflammation and injury of the squamous esophageal epithelium and an increased risk for developing Barrett’s esophagus (BE). BE is a premalignant condition that dramatically increases the risk of developing EAC [4]. While initially believed to involve a protective mechanism of trans-differentiation of squamous cells to columnar cells, more recently, it has been shown that BE arises from transitional basal cells at the squamous–columnar junction that increase in number due to GERD [5].

The EAC cascade is multifactorial, developing from a complex interplay of host anthropomorphic factors, host genetic and epigenetic factors, host immune response, as well as environmental factors [6, 7]. More recently, the esophageal microbiome has been proposed as an etiological agent that influences esophageal sphincter activity [8] and drives inflammation in the early stages of the cascade [3]. Early studies on the esophageal microbiome showed that the dominant taxon within the healthy esophagus was *Streptococcus* [9] and that the EAC cascade is characterized by a shift towards a dominance of Gram-negative bacterial species [10]. While later studies have not been able to replicate these findings [11], others have shown enrichment of specific Gram-negative bacterial species such as *Campylobacter* and *Fusobacterium* in the EAC cascade [12, 13].

Despite this, our relative understanding of the esophageal microbiome and its function in the host remains limited when compared to the gut microbiome. Here, we comprehensively assessed the esophageal microbiome of 106 prospectively recruited patients using shotgun as well as amplicon sequencing and associated specific microbial signatures with host genetics and disease.

## Results

### The esophageal microbiome clusters into community types

**Fig. 1:**
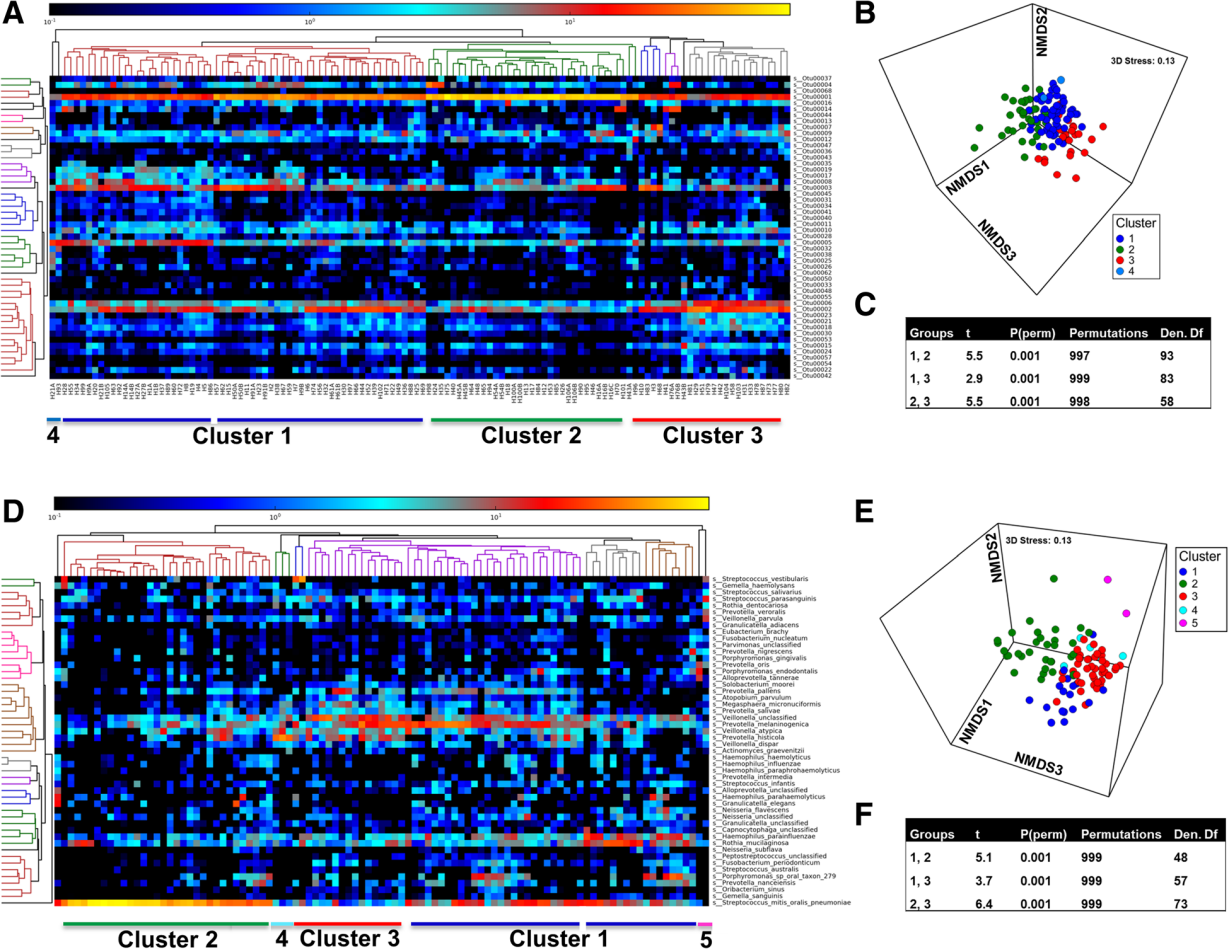
The esophageal microbiome clusters into different community types (esotypes). **a** Heatmap of relative abundances of the top 50 OTUs generated through 16S rRNA amplicon sequencing were used for a hierarchical cluster analysis (HCA) within MetaPhlan2. Dark blue to yellow correspond to 0.1–100% abundance. All available samples (*n* = 122) were utilized in this analysis. Taxonomy of OTUs is provided in Additional file 1.1. **b** Non-metric multidimensional scaling (nMDS) plot of Bray–Curtis resemblance generated from square root-transformed OTU relative abundances (all OTUs). OTU relative abundances were generated from 16S rRNA amplicon sequencing. Clusters from the HCA (panel **a**) were overlayed onto the nMDS plot. **c** PERMANOVA across HCA clusters of Bray–Curtis resemblance generated from square root-transformed OTU relative abundances. Test of the homogeneity of multivariate dispersions within groups at OTU level using PERMDISP showed no differences across clusters. ANOSIM generated a sample statistic of 0.42 and *P* = 0.001. **d** Relative abundances of the top 50 species generated through shotgun sequencing and MetaPhlan2 analysis were used for HCA. HCA was performed in MetaPhlan2. **e** nMDS plot of Bray–Curtis resemblance generated from square root-transformed species relative abundances (shotgun). All available shotgun samples were utilized in this analysis. Clusters from HCA of shotgun data (MetaPhlan2) were overlayed onto the nMDS plot. **f** PERMANOVA across shotgun HCA clusters of Bray–Curtis resemblance generated from square root-transformed species relative abundances. ANOSIM at the species level generated a sample statistic of 0.54 and *P* = 0.001

The esophageal microbiota was first profiled in brushing samples by 16S rRNA amplicon sequencing. Hierarchical clustering analysis based on the top 50 OTUs clustered samples into at least three community types (Fig. 1a, Additional file 1.1). A fourth cluster was also seen (Fig. 1a) but was not examined further due to the low number of subjects within it (*n* = 2). The three main community types were detected when OTU relative abundances were analyzed with a Bray–Curtis dissimilarity matrix (Fig. 1b), with adonis showing a significant difference between clusters (*R*^2^ = 0.16, *P* = 0.00033). The difference was further confirmed with ANOSIM (sample statistic = 0.42 and *P* = 0.001). Pairwise comparisons at the OTU level using PERMANOVA showed that each cluster differed in composition to the other (Fig. 1c), and this was confirmed up to the phylum level (Additional file 1.2).

To account for the effect of disease, the microbial composition of clusters was tested in the subset of subjects with a normal esophagus. Significant differences were still observed across the three clusters (*t* = 2.3, 2.0, 3.1; *P* = 0.001, permutations = 998, df = 41, 43, 28). Further, replicate samples from the same individuals (samples labeled A and B) clustered in the same community type despite being sampled from different locations in the lower esophagus (one from inflamed region and another from a region adjacent) (Additional file 1.3).

### Esophageal community types can be detected using shotgun sequencing

To examine the presence of community types more comprehensively, shotgun sequencing was then performed on 99 samples. Despite collecting brushing samples which limit the levels of host DNA relative to microbial DNA, a high level of contamination with human reads was still observed in our test run (Additional file 2: Figure S1A). An enrichment of microbial DNA was then performed and a 4.2 ± 1.9 (mean fold change ± SD) increase in microbial reads was established (Additional file 2: Figure S1A). No significant shifts in microbial composition were observed between the original and enriched samples (Additional file 2: Figure S1B–D); however, it is worth noting that the sequencing of original samples had a low number of microbial reads (118,202–331,573 microbial reads), potentially resulting in a less accurate profiling of the microbiome.

Taxonomic analysis was performed using MetaPhlan2 following removal of human reads with Deconseq and hierarchical clustering on the relative abundances identified three large community types (Fig. 1d). Two additional small clusters (4 and 5) were also seen (Fig. 1d) but included a very low number of subjects and were not examined further. The presence of the three main community types was also found using nMDS on a Bray–Curtis dissimilarity matrix from the MetaPhlan2 output (Fig. 1e) with ANOSIM at the species level showing a sample statistic of 0.54 and *P* = 0.001. Pairwise differences in composition among clusters were shown at species level using PERMANOVA (Fig. 1f) and confirmed up to the phylum level (Additional file 1.2). The three main clusters (1, 2, and 3) were found to be highly concordant with the amplicon sequencing (Additional file 1.3).

To ensure clustering was not due to the data analysis method, hierarchical clustering analysis was performed on taxonomic output from MEGAN6 (Additional file 1.4). This showed similar clustering to that seen from the MetaPhlan2 output. The shotgun clusters were also overlayed onto a weighted UniFrac distance matrix generated from the amplicon data (Additional file 1.5), which indicated the clustering did not result from the type of resemblance matrix used.

### Relative abundances of *Streptococcus* and *Prevotella* define the community types

**Fig. 2:**
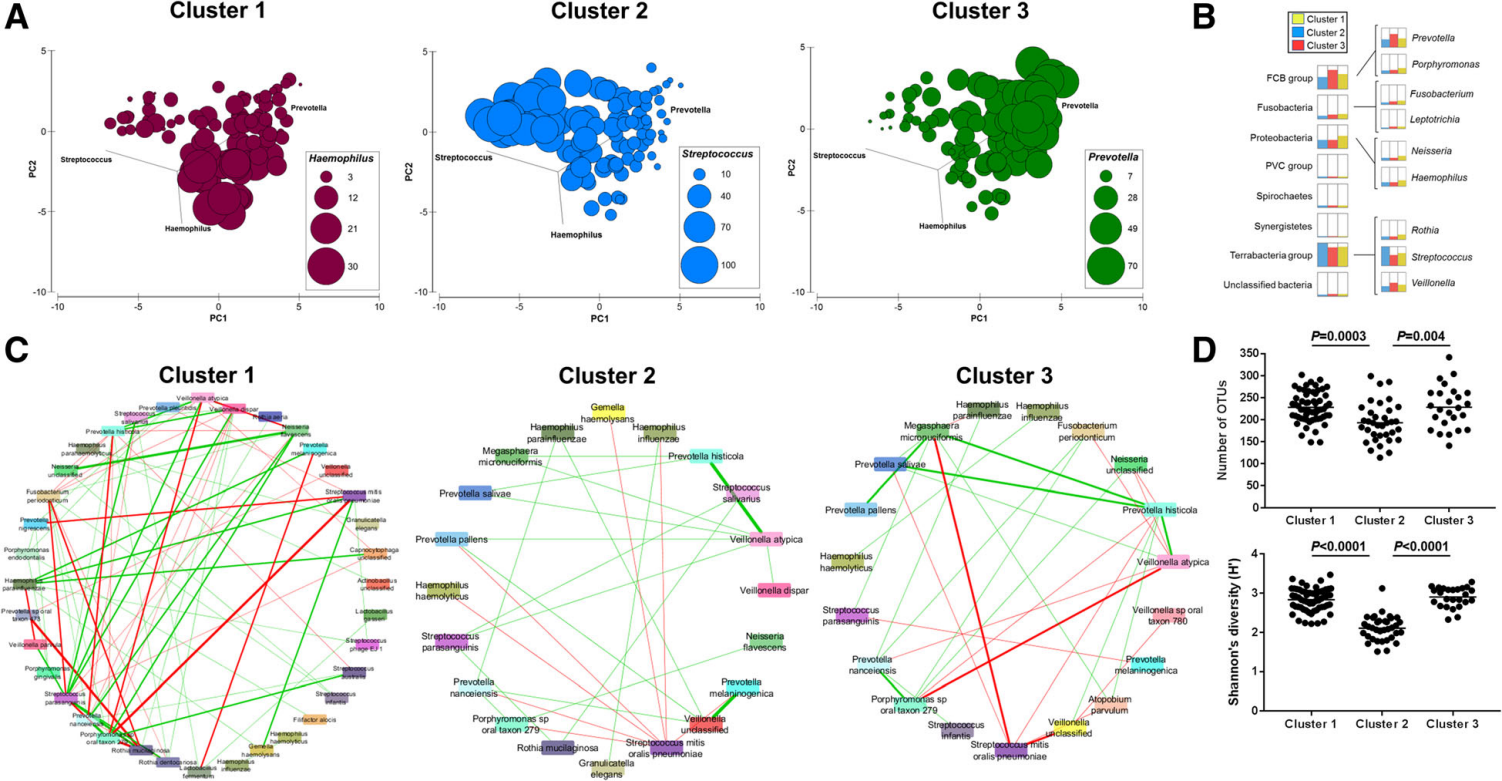
Esophageal microbiome community types are defined by diversity and composition. **a** Principal component analysis of square root-transformed OTU relative abundances. The relative abundances of *Haemophilus*, *Streptococcus*, and *Prevotella* per subject were overlayed onto the PCA to define each cluster. Size of circle corresponds to relative abundance (%) of taxon. All available samples were utilized in this analysis. **b** Comparison analysis of phylum and genus relative abundances (%) generated from MEGAN6 according to the community types. Cluster 1, yellow; cluster 2, blue; cluster 3, red. Cluster 1 showed an enrichment of *Prevotella* and *Haemophilus*, cluster 2 showed an enrichment of *Streptococcus*, and cluster 3 showed an enrichment of *Prevotella* and *Veillonella*. **c** Correlations across species (shotgun MetaPhlan2) for each community type were calculated using SparCC and correlations greater than 0.2 or lower than − 0.2 were visualized using Cytoscape. Thickness of line reflects the strength of correlation and color reflects direction (green: positive; red: negative). A complete list of SparCC correlations within each cluster is provided in Additional file 1.9. **d** Alpha diversity measures for each community type. ANOVA with Tukey’s multiple comparison tests were used to calculate *P* values. Results related to species evenness is provided in Additional file 1.10

To identify taxonomic signatures unique to each community type, analyses were performed on the 16S rRNA amplicon (Fig. 2a, Additional file 1.6) and shotgun datasets (Fig. 2b; Additional file 1.7). There were clear distinctions among the community types, with cluster 2 being dominated by *Streptococcus* (*Streptococcus mitis/oralis/pneumoniae*), cluster 3 by *Prevotella* (*Prevotella melaninogenica* and *Prevotella pallens*), and to a lesser extent *Veillonella* (Fig. 2a, b; Additional file 1.6 and 7). Cluster 1 was an intermediate type with respect to abundances of *Streptococcus* and *Prevotella* but had increased levels of *Haemophilus* (*Haemophilus parainfluenzae*) and *Rothia* (*Rothia mucilaginosa*) (Fig. 2b; Additional file 1.7).

Dirichlet multinomial mixture modeling was performed to confirm the distribution of relative abundances using unsupervised methods. At three partitions, the models were concordant with the three main clusters (89% accuracy). The relative abundance of OTUs across the three Dirichlet multinomial mixture partitions was similar to the above taxonomic classification of the clusters (Additional file 1.8). Discordant assignments arose from the misclassification of samples from the intermediate cluster (cluster 1) into either cluster 2 or 3.

SparCC analysis was then conducted on relative abundances from shotgun data to examine the relationships across individual species in each of the three community types. While the density and direction of interactions differed in each cluster, the interaction between *Streptococcus mitis/oralis/pneumoniae* and *Prevotella* spp. was consistently found to be a co-exclusion interaction (Fig. 2c; Additional file 1.9). The above findings would suggest that the ratio of *Streptococcus* to *Prevotella* is an important defining characteristic across esophageal community types.

Additional characteristics differentiating the community types were alpha diversity measures. In particular, cluster 2 could be differentiated from the other two clusters, having significantly lower levels of species richness, evenness, and Shannon’s diversity (Fig. 2d; Additional file 1.10). The dominance of *Streptococcus* in cluster 2 was found to be associated with the lower alpha diversity measures as compared to the other community types.

### Esophageal community types show distinct functional signatures

**Fig. 3:**
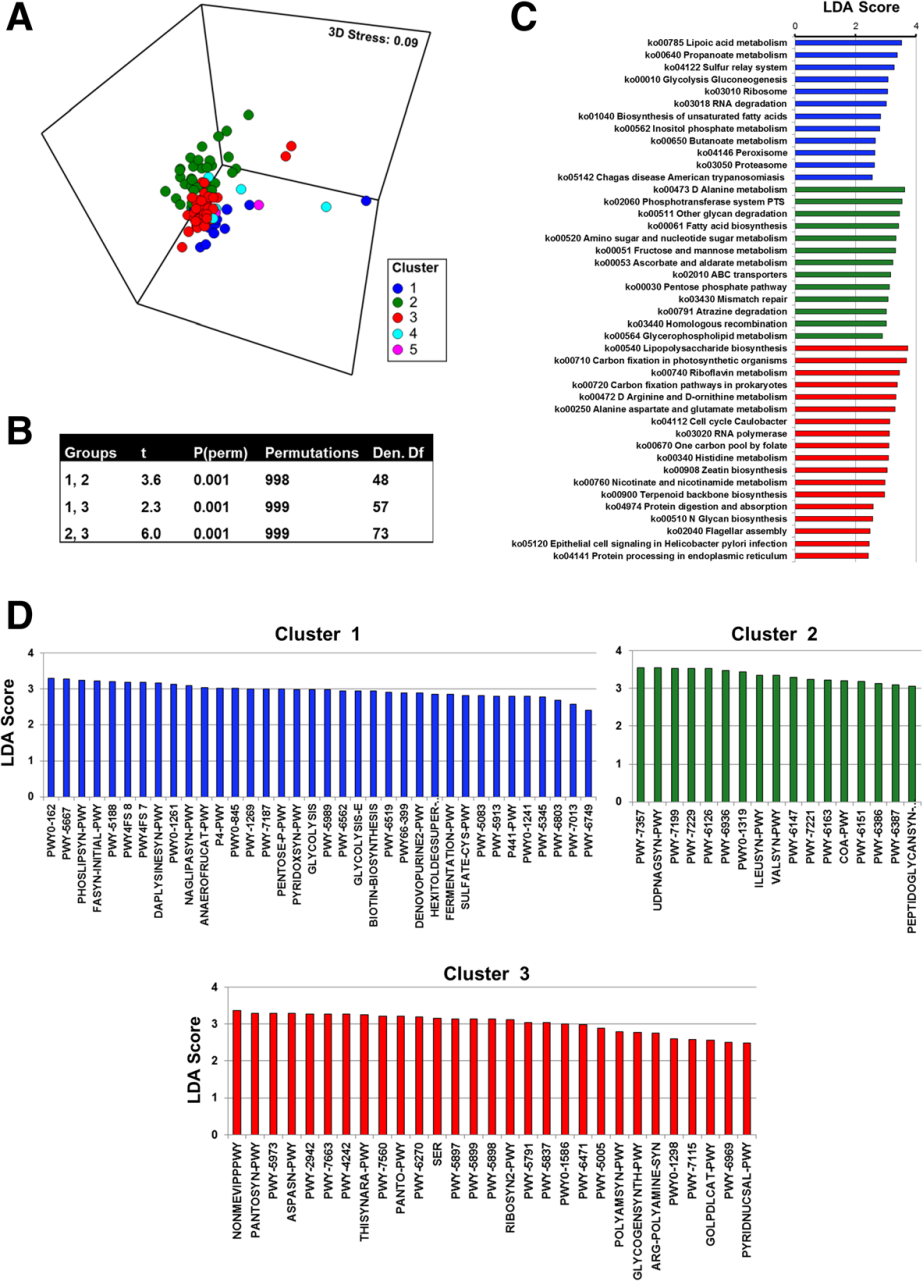
Esophageal community types are functionally distinct. **a** nMDS plot of Bray–Curtis resemblance generated from square root-transformed KEGG pathway (level 3) relative abundances (generated using HUMAnN2). Clusters from HCA of shotgun data (MetaPhlan2) were overlayed onto the nMDS plot. All available samples were utilized in this analysis. **b** PERMANOVA across shotgun HCA clusters of Bray–Curtis resemblance generated from square root-transformed KEGG pathway relative abundances. ANOSIM at KEGG pathway level 3 generated a sample statistic of 0.46 and *P* = 0.001. **c** KEGG pathways identified using LEfSe analysis to be differentially abundant across each community type. All available samples within each cluster were utilized in this analysis. Blue, cluster 1; green, cluster 2; red, cluster 3. **d** MetaCyc pathways identified using LEfSe analysis to be differentially abundant across each community type. A full list of pathway names can be found in Additional file 1.14. All available samples within each cluster were utilized in this analysis. ANOSIM for MetaCyc pathways generated a sample statistic of 0.41 and *P* = 0.001. Blue, cluster 1; green, cluster 2; red, cluster 3

Shotgun sequencing data were then analyzed using the HUMANn2 pipeline and, changes across the KEGG pathway and MetaCyc pathway reference databases were examined to determine the functional changes within the esophageal microbiome. The taxonomic community types were functionally different across both KEGG and MetaCyc (Fig. 3a; Additional file 1.11), with ANOSIM showing test statistics > 0.4 and *P* = 0.001. Pairwise differences between clusters were confirmed with PERMANOVA (Fig. 3b; Additional file 1.11C). This was also the case for the metagenomics predicted by PICRUSt from the 16S amplicon sequencing (Additional file 1.12). To determine the pathways that were enriched within each community type, LEfSe analyses were performed. A range of pathways were found to be significantly increased in each of the three community types (Fig. 3c, d; Additional file 1.13 and 14). Cluster 1 was found to be enriched for glycolysis as well as pathways involved in the metabolism of short chain fatty acids, while cluster 2 was enriched for the pentose phosphate pathway as well as fructose and mannose metabolism (Fig. 3c; Additional file 1.13). Of particular interest, cluster 3 was enriched for lipopolysaccharide biosynthesis.

### Bacterial signatures and functions are associated with age, disease, and proton pump inhibitor (PPI) usage

The contribution of the subject and clinical metadata (Table 1) to the clustering of the esophageal microbiome, as well as the overall influence of these factors, were then examined.

**Table 1:** Clinical diagnosis and symptoms of subjects recruited into the study

Disease	Number (%)	Age (years)	Gender (M)	Reflux symptoms (Y)	PPI (Y)
Normal	59 (55.7)	53.1 ± 6.9	21	1	32
GERD	29 (27.4)	52.0 ± 2.5	8	28	9
GM	7 (6.6)	59.3 ± 5.8	6	6	4
BE	5 (4.7)	59.2 ± 3.9	4	3	5
EAC	1 (0.94)	68	1	1	0
EoE	1 (0.94)	49	1	0	0

Clinical status of four subjects could not be identified due to the disintegration of the histology biopsy

*GERD*:gastroesophageal reflux disease, *GM* glandular mucosa, *BE* Barrett’s esophagus, *EAC* esophageal adenocarcinoma, *EoE* eosinophilic esophagitis, *M* male, *Y* yes, *PPI* proton pump inhibitor

#### Age

A strong effect of age on the global composition of the esophageal microbiota was found in the amplicon sequencing data (pseudo-*F* = 2.5; *P* = 0.005, df = 120) and replicated in the shotgun data (pseudo-*F* = 2.5; *P* = 0.007, df = 97). Microbial taxa associated with age were examined using DistLM (Additional file 3.1). Notably, age was positively correlated with the relative abundance of *Streptococcus* spp. such as *Streptococcus parasanguinis* but not *Streptococcus mitis/oralis/pneumoniae*. In contrast, it was inversely correlated with *P. melaninogenica* but not *P. pallens*, suggesting that age may contribute to but not fully explain the different esophageal microbial community types. Similar to the taxonomic composition, a significant effect of age (KEGG: pseudo-*F* = 3.7; *P* = 0.004, df = 97; MetaCyc: pseudo-*F* = 3.6; *P* = 0.007, df = 97) on microbiome function was found. Specifically, age had a significant influence on levels of bacterial nucleotide biosynthesis pathways (Additional file 3.2).

#### Disease

**Fig. 4:**
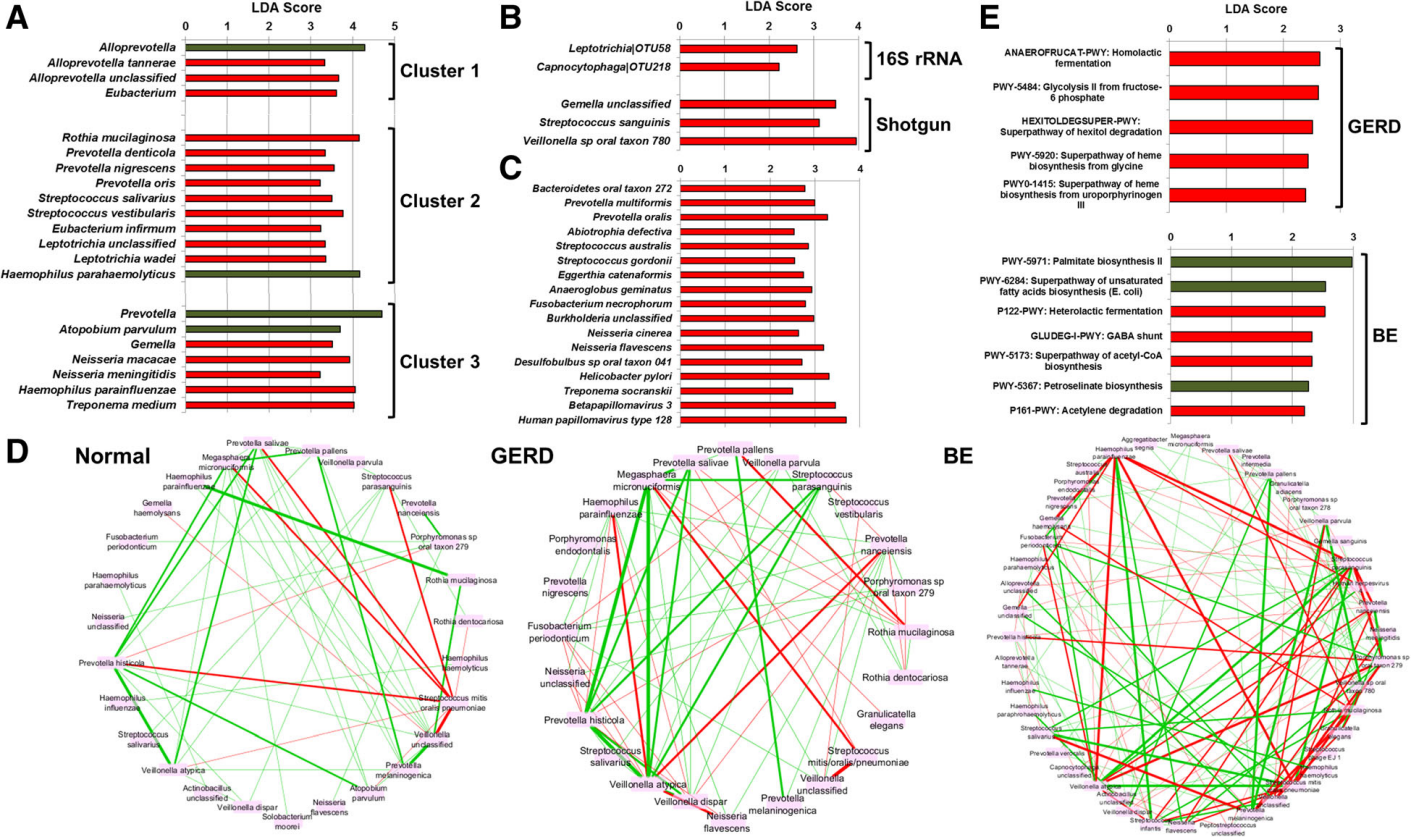
Esophageal microbial signatures associated with the early stages of the esophageal adenocarcinoma cascade. **a** Microbial taxa identified using LEfSe analysis to be differentially abundant between GERD and subjects with a normal esophagus. The analysis was performed after stratifying the subjects according to community types. Green, normal; red: GERD. **b** Microbial taxa identified using LEfSe analysis to be differentially abundant between BE and subjects with a normal esophagus. Only samples designated as BE-Y (not BE-N or BE-GERD) were employed for this analysis. Red, BE. **c** Microbial taxa identified using LEfSe analysis to be differentially abundant between GM and subjects with a normal esophagus. Samples designated as GM-Y (not GM-N or GM-GERD) were employed for this analysis. Red, GM. **d** Correlations across species (shotgun MetaPhlan2) for each disease type were calculated using SparCC and correlations greater than 0.2 or lower than − 0.2 were visualized using Cytoscape. The thickness of line reflects the strength of the correlation, while color reflects direction (green, positive; red, negative). Samples designated as BE-Y were employed for this analysis. A complete list of SparCC correlations within each disease subgroup is provided in Additional file 3.8. **e** MetaCyc pathways identified using LEfSe analysis to be differentially abundant between disease type (GERD or BE) and subjects with a normal esophagus. Only samples designated as BE-Y were employed for this analysis. Green: normal; red: disease (GERD or BE)

Disease did not have a significant impact on alpha diversity measures (Shannon’s diversity: H: 2.65 ± 0.06; GERD: 2.73 ± 0.08; GM: 2.66 ± 0.16; BE: 2.55 ± 0.21) or on global taxonomic composition (Additional file 3.3). However, an increase in a range of Gram-negative bacterial taxa was observed across the early stages of the EAC cascade. Taxa enriched in disease and never found to be enriched in normal subjects in any comparison included *Leptotrichia* (e.g., *L. wadei*), *Fusobacterium* (e.g., *F. necrophorum*), *Rothia* (e.g., *Rothia mucilaginosa*), *Campylobacter*, and *Capnocytophaga* (Fig. 4a–c; Additional file 3.4–7). Of interest, when stratified, each esophageal community type was found to gain different bacterial taxa in GERD (Fig. 4a; Additional file 3.6 and 7), suggesting that they may behave differently in disease. Further, samples collected from GM and BE patients within the area of inflammation showed no significant differences to samples collected from the region adjacent to it (PERMANOVA; *P* = 0.96 and LEfSe; no taxa identified).

SparCC analysis was repeated with subjects stratified according to disease (normal, GERD, GM, and BE) to examine the relationships across individual species in each stage of disease (Fig. 4d; Additional file 3.8). The co-exclusion relationship between *Streptococcus mitis/oralis/pneumoniae* and *Prevotella* spp. was maintained across all disease stages. Markedly denser microbial networks (i.e., a substantial increase in relationships that passed the cutoff of *r* > 0.2 or less than − 0.2 and *P* < 0.05) were observed with progression of disease stage (normal: *n* = 49 correlations, GERD: *n* = 65, BE: *n* = 122; Fig. 4d).

No effects of disease on global microbial function were observed (Additional file 3.9); however, changes in relative abundance of individual pathways were identified (Fig. 4e, Additional file 3.10). An enrichment of the bacterial superpathway of hexitol degradation was found in GERD and GM, even when accounting for community types and PPI usage. Importantly, microbial lactic acid production was increased in GERD and BE relative to subjects with a normal esophagus, with pathways such as homolactic fermentation enriched in GERD and heterolactic fermentation increased in BE. Further, an increase in heme biosynthesis from glycine or uroporphyrinogen was found in GERD as compared to subjects with a normal esophagus.

#### PPI usage and gender

Despite a small drop in species richness in subjects with a normal esophagus on PPIs as compared to subjects with a normal esophagus without PPIs (number of OTUs: 208 ± 7 vs 221 ± 6, *P* = 0.15), gender and PPI usage did not have a significant impact on alpha diversity measures or on the global taxonomic composition of the esophageal microbiota (Additional file 2: Figure S2A–F). When patients were stratified by disease, a minor effect of PPI on microbial composition was observed in the GERD patients for amplicon sequencing (Additional file 2: Figure S2E); however, the strength of this effect was not replicated in the shotgun data (Additional file 2: Figure S2F). In line with the small effect of PPI on microbial composition of GERD subjects but not subjects with a normal esophagus, a higher number of individual bacterial taxa were found to be associated with PPI usage in GERD patients as compared to subjects with a normal esophagus (Additional file 2: Figure S3A, B).

No effects of gender or PPI usage on global microbial function were observed (Additional file 2: Figure S4A–F). Changes in relative abundance of individual pathways were identified for PPI usage but not gender (Additional file 2: Figure S5A, B). Increases in pathways TCA cycle VII (acetate producers) as well as biosynthesis of antibiotics such as vancomycin and streptomycin were found to be increased in subjects who had taken PPIs.

### Microbial eukaryotes and viruses are present in the esophageal microbiome

**Fig. 5:**
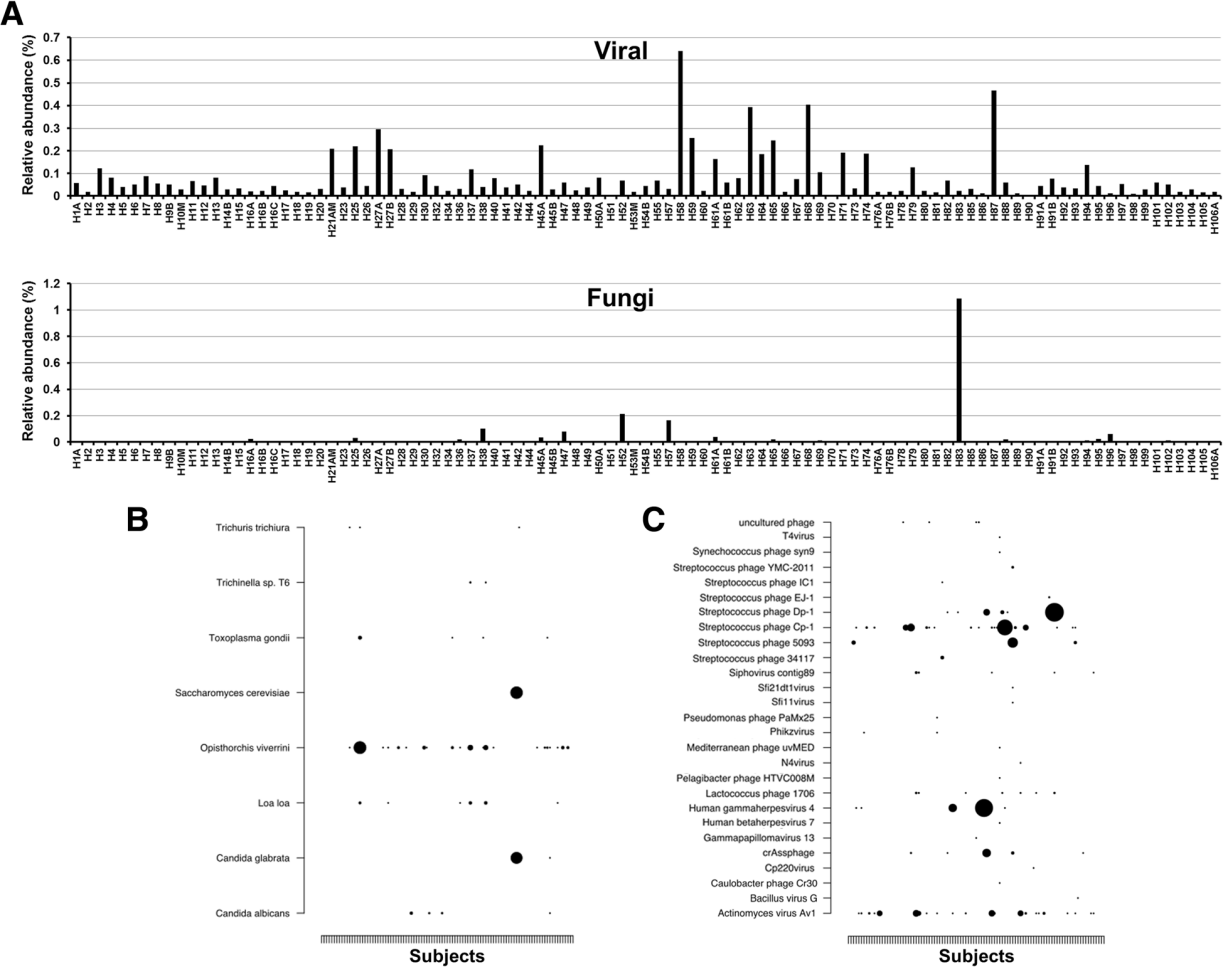
Presence of non-bacterial microbial taxa within the esophageal microbiome. **a** Relative abundance of viruses and fungi within the esophageal microbiome of each subject. Relative abundances were calculated using taxonomy arising from MEGAN6. This was used due to its capacity to detect microbial eukaryotes. **b** Relative abundances of specific eukaryotic and **c** viral taxa within each subject arising from the MEGAN6 analysis. Size of circle signifies the relative abundance levels of the organism. Size of circles ranges from 0.0094 (smallest) to 0.64% (largest) for viruses and 0.0097–1.08% for eukaryotes. Subjects are ordered by time of recruitment (left to right). The presence of *Candida* spp. and *Saccharomyces* were confirmed using 18S amplicon sequencing. We could not confirm the detection of *Trichuris*, *Trichinella*, and *Loa loa*; thus, these identifications should be taken with caution as they most probably arose from misclassifications within the metagenomic annotation process. No association with any of the clinical metadata was found

Shotgun sequencing identified a range of taxa within the esophagus belonging to microbial lineages other than bacteria (Fig. 5). These organisms had relatively low abundance as compared to the bacteriome and were not detected in all patients. Archaea classified to Halobacteria (3 patients; 0.0094, 0.0096, and 0.013%) and *Methanosarcina* (1 patient; 0.030%) were detected in 4/92 (4.3%) subjects whereas microbial eukaryotes classified to *Candida albicans*, *Candida glabrata*, *Saccharomyces cerevisiae*, and others were found (0.0097–1.08%) in 19/92 (20.6%) subjects (Fig. 5a, b). Amplicon sequencing of the 18S rRNA gene confirmed the presence of *C. albicans*, *C. glabrata*, and *Saccharomyces* in four patients that were found to have relatively higher levels (0.08–1.08%) of these organisms in the shotgun analysis. Detection of parasitic worms such as *Trichuris*, *Trichinella*, and *Loa loa* could not be confirmed using 18S rRNA gene amplification and should be taken with caution as they most probably arose from misclassifications within the metagenomic annotation process.

Viruses, in particular, phages, were more common and were identified in 90/92 (97.8%) subjects at a relative abundance that ranged from 0.0094–0.64%. The classification of the viral population identified a range of phages including *Streptococcus*, *Campylobacter*, *Lactococcus*, and *γ*-Proteobacteria phages (Fig. 5a, c). Human viruses such as betaherpesvirus 7, gammaherpesvirus 4, and gammapapillomavirus 13 were also identified in 1, 5, and 1 subjects, respectively.

### Host genetics is associated with the composition of the esophageal microbiome

**Fig. 6:**
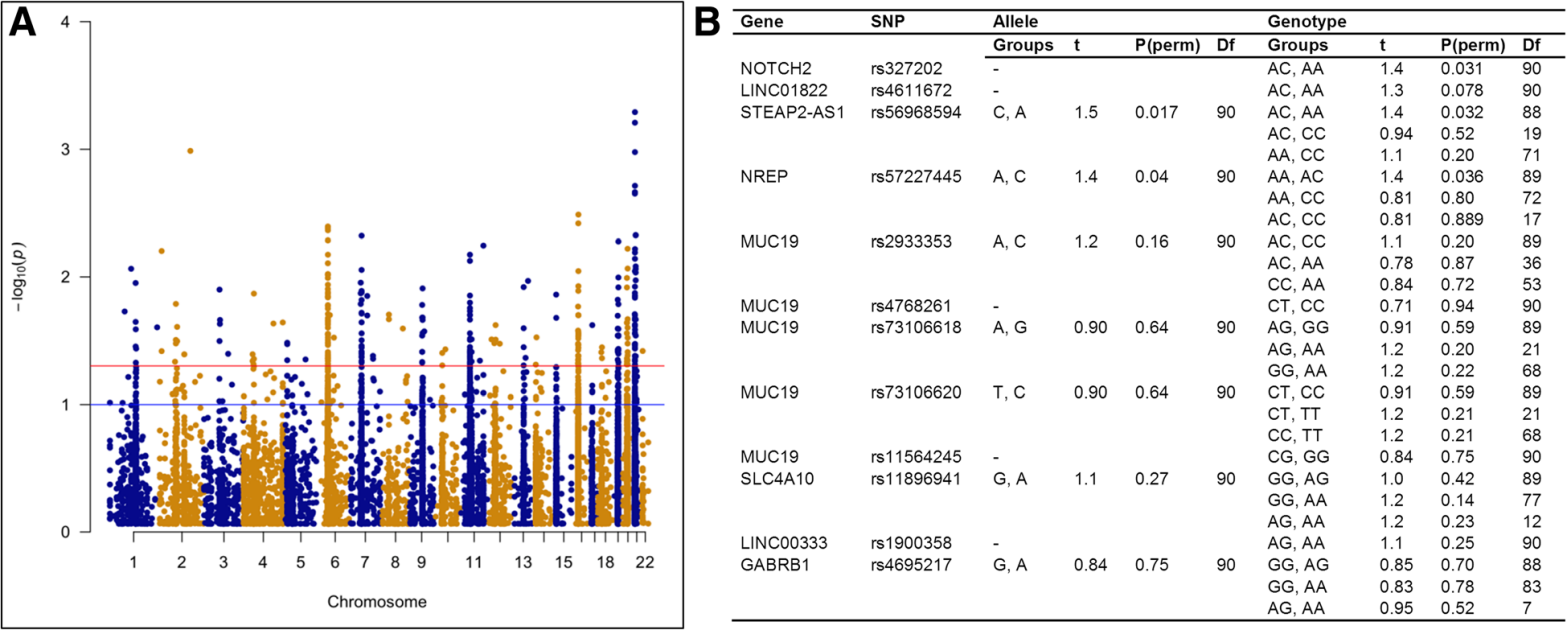
Host genetic factors associated with the esophageal microbiome. **a** Host SNPs identified using MicrobiomeGWAS to be correlated with the Bray–Curtis resemblance matrix generated from square root-transformed species relative abundances (taxonomy arising from MetaPhlan2). Human SNPs were identified using the GATK toolkit onto the shotgun sequencing reads (depth of coverage (dp) = 2; minimum number of samples = 50). Blue line represents *P* = 0.1 and red line represents *P* = 0.05; all SNPs above the red line have significant *P* values. A complete list of SNPs across different thresholds is provided in Additional file 4.1–3. SNPs associated with microbiome composition mapped across most human chromosomes suggesting the analysis was not biased by low depth of coverage of the human genome. **b** PERMANOVA on Bray–Curtis resemblance matrix generated from square root-transformed species relative abundances (taxonomy arising from MetaPhlan2). Tests were applied across allele and genotype frequencies for human SNPs validated using Fluidigm custom SNPtype assays. A table of the genotyping results generated from the Fluidigm custom assays is provided in Additional file 4.4 and 5

Despite enrichment for microbial DNA, shotgun sequencing still resulted in a substantial number of reads that mapped to the human genome (~ 70–90% reads/sample; Additional file 2: Figure S1A). The GATK toolkit was used to detect single nucleotide polymorphisms (SNPs) within our samples. MicrobiomeGWAS (tool to analyze microbiome-genome-wide association study data and identify SNPs associated with overall microbial composition) was then employed to identify SNPs associated with the Bray–Curtis resemblance matrix generated at the species level (shotgun taxonomy from MetaPhlan2). Three thresholds were used for this analysis: (1) a threshold of depth of coverage (dp) = 2, minimum number of samples = 50 (Fig. 6a, Additional file 4.1); (2) dp = 2, minimum number of samples = 75 (Additional file 4.2); (3) dp = 3, minimum number of samples = 75 (Additional file 4.3). The analysis identified SNPs associated with microbiome composition across most human chromosomes (Fig. 6a) suggesting it was not biased by our low depth of human genome coverage.

Nineteen SNPs were selected for validation using custom Fluidigm SNP Type assays. These included 14 SNPs within *BAGE2*, *FRG1BP*, *MUC19*, *SLC4A10*, *LINC00333*, *ANKRD36*, *NOTCH2*, *LINC01822*, *GABRB1*, *GYPB*, *STEAP2-AS1*, *NREP*, *NCOR1P1*, *PRIM2*, *OR4C45*, *GUSBP1*, and *LOC102723769* (Additional file 4.4). A further 5 previously defined SNPs within *MUC19* (Additional file 4.4) were included due to the relevance of this gene within the gastrointestinal tract. Three host SNPs [*NOTCH2* rs327202, *STEAP2-AS1* rs56968594, and *NREP* (P311) rs57227445] were validated in the allele and genotype analyses using PERMANOVA (Fig. 6b; Additional file 4.5). SNPs in *LINC01822* (rs4611672; *P* = 0.078) and *MUC19* (rs2933353; *P* = 0.16) did not reach significance in the validation studies (Fig. 6b; Additional file 4.5).

## Discussion

Little is known about the esophageal microbiome relative to our understanding of the composition and function of the gut microbiome. Here, the esophageal microbiome was comprehensively assessed using 16S and 18S rRNA amplicon sequencing as well as shotgun metagenomics in esophageal brushings of a cohort of prospectively recruited subjects. The esophageal microbiome was found to cluster into community types termed “esotypes.” The defining characteristic of the esotypes was the dominant organisms, with one type being dominated by *S. mitis/oralis/pneumoniae*, the other *P. melaninogenica*, *P. pallens*, and *Veillonella*, and the third *Prevotella, H. parainfluenzae*, and *R. mucilaginosa*. However, another differentiating factor was microbial richness and evenness, with the esotype enriched for *Streptococcus* having lower levels than the other two esotypes. A co-exclusion relationship between the two dominant taxa *Streptococcus* and *Prevotella* was consistently observed in the esophageal microbiome, which appears to drive the formation of the community types. There is precedent in the literature for the clustering of the microbiome into community types with the lower gut microbiome now accepted to cluster into enterotypes [14, 15] and the vaginal microbiome clustering into several vaginotypes [16]. There is also a similar antagonistic relationship reported between two of the dominant taxa within the gut microbiome *Bacteroides* and *Prevotella* [17]. Further evidence to support our conclusions comes from the finding that samples from different locations in the lower esophagus of the same individual consistently clustered into the same esotype regardless of inflammation or disease.

The identified esotypes were found to be functionally distinct. One cluster was found to be enriched for glycolysis as well as pathways involved in the metabolism of short chain fatty acids, while another was enriched for the pentose phosphate pathway as well as fructose and mannose metabolism. Notably, one community type was enriched for lipopolysaccharide biosynthesis pathways. Given that lung microbiome types enriched for *Prevotella* and *Veillonella* have been found to regulate the basal inflammatory status in the mucosa [18], it would be of interest to determine if higher levels of LPS impacts basal inflammatory levels in the esophagus.

Subject age significantly influenced microbial composition in the esophagus. While effects of age on the abundances of *Streptococcus* spp. and *Prevotella* spp. were identified, age did not appear to influence *S. mitis/oralis/pneumoniae* abundance, one of the more abundant *Streptococcus* taxa. This suggested that age may contribute to the development of esotypes but not fully explain their presence in the cohort. The influence of age on the esophageal microbiome is to be expected given that it influences the composition of the oral and gut microbiota [19–21]. PPI usage was found to have a mild effect on the esophageal microbiome. PPIs are known to significantly impact the lower gut microbiota [22], likely due to decreasing acid levels in the stomach. The effect of PPI on the esophageal microbiota may also be related to acid levels given that the effect was stronger in GERD patients as compared to subjects with a normal esophagus. Of interest, PPI usage appeared to increase the levels of bacterial antibiotic production pathways which may also contribute to shaping the composition of the microbiome post-PPI therapy.

Global shifts in the esophageal microbiome were not observed across the early stages of the EAC cascade. However, an enrichment of a wide range of Gram-negative bacterial taxa commonly associated with the oral cavity was found in GERD, GM, and BE when compared to subjects with a normal esophagus. These included *Leptotrichia*, *Fusobacterium*, *Rothia*, *Campylobacter*, and *Capnocytophaga*. This would suggest that disease may be associated with an enrichment of a diverse set of organisms with common properties rather than specific organisms and is supported by the finding that each esotype responded differently during GERD. The enrichment of networks of oral bacteria taxa have been shown in gastric cancer [23] and colorectal cancer [24] and have been linked to inflammation in the gut [25]. Further, lactic acid production pathways (homolactic and heterolactic fermentation) were increased in the EAC cascade. Dysregulated lactate metabolism is one of the hallmarks of carcinogenesis [26]; thus, the microbial contribution towards lactate availability and how that impacts host cells should be explored further, given that a similar increase in lactate-producing bacteria has been reported in gastric adenocarcinoma [23].

A two-step process of detecting host SNPs associated to microbiome composition using the shotgun data followed by validation using custom SNP type assays identified three host SNPs (*NOTCH2* rs327202, *STEAP2-AS1* rs56968594, and *NREP* rs57227445) to be significantly associated with microbial composition in the esophagus. An additional two SNPs, *LINC01822* rs4611672 and *MUC19* rs2933353, were found to have borderline relationships. The association with SNPs in *NREP* and *NOTCH2* would suggest that the transforming growth factor β1 (TGF-β1) [27, 28] and Notch signaling pathways are linked to the esophageal microbiome, which is of interest given that both are involved in the EAC cascade [3]. It is currently unclear what role *STEAP2-AS1* plays in the host, but *STEAP2* is a metalloreductase involved in iron and copper uptake and reduction, and has been linked to fatty acid metabolism as well as the response to inflammation [29]. While the association with *MUC19* rs2933353 was not significant, given that MUC19 is a salivary mucin that plays a role in pathogen clearance in the oral cavity [30] and SNPs in this gene are associated with Crohn’s disease [31], further validation of this association in a larger cohort would be required.

Non-bacterial members of the esophageal microbiome were detected in our cohort. A low prevalence of archaea was detected in our cohort. Fungi such as *C. albicans*, *C. glabrata*, and *S. cerevisiae* and others were found in ~ 20% of patients in low abundance, while the identified virome consisted mainly of bacteriophages that putatively target the resident bacteriome, with the exception of human DNA viruses like betaherpesvirus, gammaherpesvirus, and gammapapillomavirus detected in some patients. These findings would suggest that a more specific characterization of the mycobiome with ITS amplicon sequencing and better methods to extract the virome would be required in the future to properly assess the contribution of these microbial lineages to the esophageal microbiome.

Of relevance to the field, a novel strategy was employed in this study to mucosal microbial communities using shotgun metagenomics. First, given that mucosal brushings and biopsies have been shown to provide similar assessments of the microbiome [32], brushings were collected to limit the levels of host DNA. Extracts were then effectively enriched for microbial DNA, which provided a significant improvement in the ratio of human to microbial reads. This strategy allowed us to successfully profile the mucosal microbiome in the esophagus.

This study is not without limitations. While blinded prospective recruitment can decrease bias during recruitment of subjects and improve patient classification, it can result in uneven group sizes, specifically smaller group sizes for rarer conditions. This can influence statistical power for comparisons across these groups. Further, the microbial signatures identified are based on relative abundances. Quantitative data using qPCR will allow for better validation of the presence of esotypes as well as signatures associated with the disease.

## Conclusions

The esophageal microbiome was found to cluster into functionally distinct community types (esotypes) defined by *Streptococcus* and *Prevotella,* suggesting that these community types need to be accounted for similar to what has been suggested for the gut microbiome [15]. While age was found to be a significant factor driving microbiome composition, relevant bacterial signatures and functions associated with the early stages of the EAC cascade were identified. The presence of non-bacterial microbes in the esophageal microbiome was reported and should be profiled in more detail in the future. Importantly, specific host SNPs in genes previously associated with EAC were found to be associated with the composition of the esophageal microbiome.

## Methods

### Patient recruitment and collection of specimens

One hundred six predominantly Caucasian subjects who underwent upper gastrointestinal endoscopy at the Prince of Wales Hospital (Sydney) for examination of their gastrointestinal symptoms were recruited prospectively (Table 1; Additional file 5.1). Subjects who had a normal esophagus by histological assessment were considered controls. These subjects may be prescribed PPIs due to some mild reflux symptoms; however, no reflux esophagitis is detected through histology. Patients found with glandular mucosa but without any presence of intestinal metaplasia were classified within the GM group. Subjects who had been prescribed antibiotics or non-steroidal anti-inflammatory drugs (NSAIDs) in the 2-month period prior to recruitment were excluded. A set of two samples were collected at endoscopy, an oesophageal brushing for assessment of the microbiome and an oesophageal biopsy from the same location as the brushing for histological analysis to be conducted by the pathology services at Prince of Wales Hospital. The brushing to assess the microbiome was collected first, and extreme care was taken to ensure the non-reusable brush was not contaminated by saliva. In patients where possible Barrett’s esophagus was suspected after visual examination, an additional set of samples were collected from a region adjacent to the metaplastic region. Information pertaining to gender, age, and medication use was obtained from the medical records of each subject at the time of endoscopy. Researchers were blinded to the results of the histological analysis until sequencing was completed. Ethics approval was obtained from the South Eastern Sydney Local Health District Human Research Ethics Committee (HREC 13/375 and HREC 16/020). All subjects recruited to the study signed a written informed consent, and all experiments were performed in accordance with relevant guidelines and regulations.

### DNA extraction, 16S rRNA and 18S rRNA amplicon sequencing, and data analysis

DNA was extracted from esophageal brushings using Gentra Puregene Tissue kit (Qiagen) according to the manufacturer’s instructions. The 16S rRNA gene was amplified using the KAPA HiFi HotStart ReadyMix (95 °C for 3 min, 25 cycles of 95 °C for 30 s, 55 °C for 30 s, 72 °C for 30 s, followed by a final step of 72 °C for 5 min) and the earth microbiome primers (515F-806R). Indices and Illumina sequencing adapters were attached using the Nextera XT Index Kit, and sequencing was performed with Illumina MiSeq 2 × 250 bp chemistry at the Ramaciotti Centre for Genomics. Raw reads were analyzed using the MiSeq standard operating procedures within mothur v1.39.1 [33, 34] with SILVA SEED 16S rRNA reference alignment and classification using RDP (read depth 30,081 clean reads/sample). PICRUSt [35] was employed to predict metagenomics from 16S rRNA gene relative abundance data following classification with the GreenGenes database [36]. Extraction controls in the form of kit buffers and PCR reagents are routinely sequenced in our lab (Additional file 2: Figure S6A) and show no overlap with our samples. An empty brushing sample extracted and sequenced among our 16S amplicon sequencing samples (sample: H76EX in ENA submission) showed high levels of *Ralstonia* and *Bradyrhizobium* and a very minimal amount of carry-over from other samples sequenced on the same run (Additional file 2: Figure S6B).

The 18S rRNA gene was amplified using the 5PRIME HotMasterMix (94 °C for 3 min, 35 cycles of 94 °C for 45 s, 57 °C for 60 s, 72 °C for 90 s, followed by a final step of 72 °C for 10 min) and the primers (1391f-EukBr). Indices and Illumina sequencing adapters were attached using the Nextera XT Index Kit, and sequencing was performed with Illumina MiSeq 2 × 150 bp chemistry. Raw reads were analyzed using the MiSeq standard operating procedures within mothur v1.39.1 with SILVA SEED v123 reference alignment and classification using Protist Ribosomal Reference database (PR2). The resulting data matrices were used for analysis (read depth 22,238 clean reads/sample).

Both 16S and 18S amplicon sequencing were performed on original DNA extracts from the esophageal brushings.

### Enrichment for microbial DNA and preliminary shotgun sequencing

To optimize sequencing, two separate Illumina MiSeq 2 × 250 bp chemistry runs were performed on three samples before and after enrichment of microbial DNA. Indices and Illumina sequencing adapters were attached using the Nextera XT Index Kit. Microbial DNA was enriched using NEBNext® Microbiome DNA Enrichment Kit (New England Biolabs) according to the manufacturer’s instructions.

### Shotgun sequencing and data analysis

Given the significant increase in the number of microbial reads following enrichment, DNA samples (*n* = 99, see Additional file 5.1) were enriched as above. These 99 samples were selected based on two factors: (1) relevant histology (normal, GERD, GM-Y and BE-Y, and EAC) and (2) efficient DNA enrichment. Most samples designated as − *N* were excluded to avoid duplication and ensure adequate sequencing depth. Indices and Illumina sequencing adapters were attached using the Nextera XT Index Kit, and sequencing was performed with Illumina HiSeq 2500 2 × 250 bp chemistry. Shotgun metagenomic reads were first analyzed with DeconSeq [37] for identification and filtering of human DNA sequences. Sequencing reads were assessed for quality using FastQC v0.11.2. SolexaQA was then applied to calculate sequence quality statistics and perform quality filtering. Paired-end raw reads were trimmed with the BWA trimming mode at a threshold of Q13 (*P* = 0.05) using the read trimmer module DynamicTrim. Filtered reads that were less than 50 bp in length were then discarded using LengthSort. MetaPhlAn2 [38] was employed to generate taxonomic profiles from the shotgun reads, while HUMAnN2 (HMP Unified Metabolic Analysis Network) [39] was used to determine the metabolic contributions within the samples. The HUMAnN2 pipeline involved mapping of the metagenomic reads against Uniref orthologous gene family, MetCyc UniPathway, and KEGG. MEGAN6 [40] was also used to generate microbial taxonomic and functional profiles, as well as a core esophageal microbiome. Species-level annotations should be taken with caution.

### Statistical analysis

Alpha diversity, beta diversity, and changes across individual taxons were examined for a range of available predictors. Prior to diversity comparisons, the OTU counts were rarefied to account for uneven sequencing depths among samples. α-diversity measures were calculated in mothur v1.39.1, and differences were examined using ANOVA with post hoc Tukey’s multiple comparisons test (GraphPad Prism 7). To determine differences in microbial composition, multivariate analyses such as non-metric multidimensional scaling (nMDS), permutational MANOVA (PERMANOVA), PERMDISP (tests the homogeneity of multivariate dispersions within groups), and DistLM were performed on either a Bray–Curtis resemblance matrix generated from square root-transformed relative abundances or a weighted UniFrac distance matrix. Dirichlet multinomial mixtures [41] were performed in mothur using the get.communitytype command. To identify individual taxa that differed significantly across conditions, Linear Discriminant Analysis Effect Size (LEfSe) [42] was performed across a range of co-variates including microbiome clusters, PPI usage, gender, and disease. Once identified, clusters were accounted for in LEfSe analyses for other co-variates.

### Microbial association network analysis using SparCC

Networks for disease and community types were inferred using SparCC [43] following the estimation of correlation coefficients and adjustment for compositional effects; 100 shuffles were used for the permutation-based approach in SparCC. Cytoscape v3.5.1 was employed to generate plots for significant co-occurrence and co-excluding interactions (correlation coefficients > 0.2 or less than − 0.2, *Q* < 0.05). The size and color of the nodes correspond to weighted node connectivity (WNC) scores.

### Host-microbiome correlation analysis using GATK and MicrobiomeGWAS

Individual sample reads from shotgun sequencing were mapped to the human reference using bwa v0.7.9a. This was followed by SNP identification using the GATK 3.5 genome analysis toolkit. To identify host genetic variants associated with microbiome composition, MicrobiomeGWAS [44] was used to combine the variant file (vcf) and the Bray–Curtis dissimilarity matrix of the microbial relative abundances generated from the shotgun sequencing data by MetaPhlAn2.

### Fluidigm assays

Custom SNPtype assays allow for genotyping based on allele-specific PCR SNP detection using Dynamic Array™ Integrated Fluidic Circuits. A tagged, allele-specific PCR primer and a common reverse primer are employed, as well as a universal fluorescent probe. To confirm the association between a subset of SNPs and microbiome composition, Fluidigm custom SNP Type 96.96 assays were designed (Additional file 4.4) and conducted according to the manufacturer’s instructions on a BioMark HD (Fluidigm) at the Ramaciotti Centre for Genomics. Briefly, 24 custom SNP assays targeting rs10433076, rs113646232, rs11564245, rs11896941, rs1900358, rs201169969, rs2933353, rs327202, rs4572690, rs4611672, rs4695217, rs4768261, rs5011360, rs56968594, rs57227445, rs62211564, rs62402964, rs73106618, rs73106620, rs73115384, rs75009827, rs7714828, rs79153215, and rs80014625 were submitted to Fluidigm for assay design. Plates containing custom assays were then used to genotype the samples with a maximum loading of volume for DNA. A pre-amplification step as recommended by the manufacturer was used to ensure samples with low concentrations were amplified.

## Additional files

Additional file 1: The esophageal microbiome clusters into different community types. (XLSX 632 kb)

Additional file 2: **Figure S1.** Comparison of the esophageal microbiome prior to and after enrichment for microbial reads. **Figure S2.** Effects of proton pump inhibitors and gender on esophageal microbiome composition. **Figure S3.** Esophageal microbial signatures associated proton pump inhibitor use. **Figure S4.** Effects of proton pump inhibitors and gender on functional pathways within esophageal microbiome. **Figure S5.** Esophageal microbiome functional signatures associated proton pump inhibitor use. **Figure S6.** Negative controls relevant to this study. (ZIP 3954 kb)

Additional file 3: Esophageal microbial signatures associated with host factors. (XLSX 79 kb)

Additional file 4: Host genetic variations and the esophageal microbiome. (XLSX 59 kb)

Additional file 5: Clinical metadata of subjects. (XLSX 15 kb)
