# Supplementary material for: Signatures within the esophageal microbiome are associated with host genetics, age, and disease
Source: Microbiome. 2018 Dec 17;6:227. doi: 10.1186/s40168-018-0611-4 (PMC6297961; doi:10.1186/s40168-018-0611-4)
Supplement: Supplementary file 2 — Figure S1. Comparison of the esophageal microbiome prior to and after enrichment for microbial reads. Figure S2. Effects of proton pump inhibitors and gender on esophageal microbiome composition. Figure S3. Esophageal microbial signatures associated proton pump inhibitor use. Figure S4. Effects of proton pump inhibitors and gender on functional pathways within esophageal microbiome. Figure S5. Esophageal microbiome functional signatures associated proton pump inhibitor use. Figure S6. Negative controls relevant to this study. (ZIP 3954 kb) [file 40168_2018_611_MOESM2_ESM.zip › Additional figure legends.docx]

**Additional figure legends**

**Additional figure 1**

**Comparison of the esophageal microbiome prior to and after enrichment for microbial reads.** **A:** Microbial DNA was enriched using NEBNext® Microbiome DNA Enrichment Kit. ‘M’ refers to samples post-enrichment. Number of microbial reads remaining after removal of human reads with Deconseq were H10: 331,573 reads, H10M: 2,860,879 reads, H21A: 118,202 reads, H21AM: 745,300 reads, H53: 215,193 reads, and H53M: 1,124,582 reads. **B:** nMDS plot of Bray-Curtis resemblance matrix generated from square-root transformed species relative abundances (taxonomic data from MetaPhlan2). **C:** HCA of six samples using relative abundances at the phylum level (taxonomic output using MetaPhlan2). **D:** HCA of six samples using relative abundances at the genus level (taxonomic output using MetaPhlan2). Heat maps were drawn using MetaPhlan2.

**Additional figure 2**

**Effects of proton pump inhibitors and gender on esophageal microbiome composition. A:** Non-metric multidimensional scaling (nMDS) plot of Bray-Curtis resemblance generated from square-root transformed OTU relative abundances (all OTUs). OTU relative abundances were generated from 16S rRNA amplicon sequencing. Samples were labelled according to gender. **B:** nMDS plot of Bray-Curtis resemblance generated from square-root transformed species relative abundances (shotgun). All available shotgun samples were utilized in this analysis. Samples were labelled according to gender. **C:** nMDS plot of Bray-Curtis resemblance generated from square-root transformed OTU relative abundances (all OTUs). OTU relative abundances were generated from 16S rRNA amplicon sequencing. Samples were labelled according to PPI usage. **D:** nMDS plot of Bray-Curtis resemblance generated from square-root transformed species relative abundances (shotgun). All available shotgun samples were utilized in this analysis. Samples were labelled according to PPI usage. **E:** PERMANOVA across gender and PPI usage of Bray-Curtis resemblance generated from square-root transformed OTU relative abundances. An additional sub-analysis was performed for PPI usage within subjects with a normal esophagus and subjects with GERD. **F:** PERMANOVA across gender and PPI usage of Bray-Curtis resemblance generated from square-root transformed species relative abundances. An additional sub-analysis was performed for PPI usage within subjects with a normal esophagus and subjects with GERD.

**Additional figure 3**

**Esophageal microbial signatures associated proton pump inhibitor use. A:** Microbial taxa identified using LEfSe analysis to be differentially abundant between subjects on PPIs as compared to subjects not on PPIs. Analysis was limited to subjects found to have a normal esophagus. Green: PPI No; Red: PPI Yes. **B:** Microbial taxa identified using LEfSe analysis to be differentially abundant between subjects on PPIs as compared to subjects not on PPIs. Analysis was limited to subjects found to have GERD. Green: PPI No; Red: PPI Yes. This analysis was not performed in GM or BE patients due to lack of power.

**Additional figure 4**

**Effects of proton pump inhibitors and gender on functional pathways within esophageal microbiome.** Relative abundances were generated from HUMANn2 analysis of shotgun sequencing data. **A:** nMDS plot of Bray-Curtis resemblance generated from square-root transformed KEGG pathway (level 3) relative abundances. Samples were labelled according to gender. **B:** nMDS plot of Bray-Curtis resemblance generated from square-root transformed MetaCyc pathway relative abundances. Samples were labelled according to gender. **C:** nMDS plot of Bray-Curtis resemblance generated from square-root transformed KEGG pathway (level 3) relative abundances. Samples were labelled according to PPI usage. **D:** nMDS plot of Bray-Curtis resemblance generated from square-root transformed MetaCyc pathway relative abundances. Samples were labelled according to PPI usage. **E:** PERMANOVA across gender and PPI usage of Bray-Curtis resemblance generated from square-root transformed KEGG pathway (level 3) relative abundances. **F:** PERMANOVA across gender and PPI usage of Bray-Curtis resemblance generated from square-root transformed MetaCyc pathway relative abundances.

**Additional figure 5**

**Esophageal microbiome functional signatures associated proton pump inhibitor use.** Relative abundances were generated from HUMANn2 analysis of shotgun sequencing data. **A:** Microbial KEGG pathways (level 3) identified using LEfSe analysis to be differentially abundant between subjects on PPIs as compared to subjects not on PPIs. Analysis was performed across all subjects. Green: PPI No; Red: PPI Yes. **B:** Microbial MetaCyc pathways identified using LEfSe analysis to be differentially abundant between subjects on PPIs as compared to subjects not on PPIs. Analysis was performed across all subjects. Green: PPI No; Red: PPI Yes.

**Additional figure 6**

**Negative controls relevant to this study. A:** Negative controls in the form of kit buffers and PCR reagents routinely sequenced. High levels of *Bradyrhizobium* and *Lactobacillus* were found in different controls. **B:** Empty brushing extracted and sequenced among our 16S amplicon sequencing samples (sample: H76EX in ENA submission). High levels of *Ralstonia* and *Bradyrhizobium* were detected and a minimal amount of carry-over from other samples sequenced on the same run.
