# Supplementary figures and images for: Signatures within the esophageal microbiome are associated with host genetics, age, and disease
Source: Microbiome. 2018 Dec 17;6:227. doi: 10.1186/s40168-018-0611-4 (PMC6297961; doi:10.1186/s40168-018-0611-4)

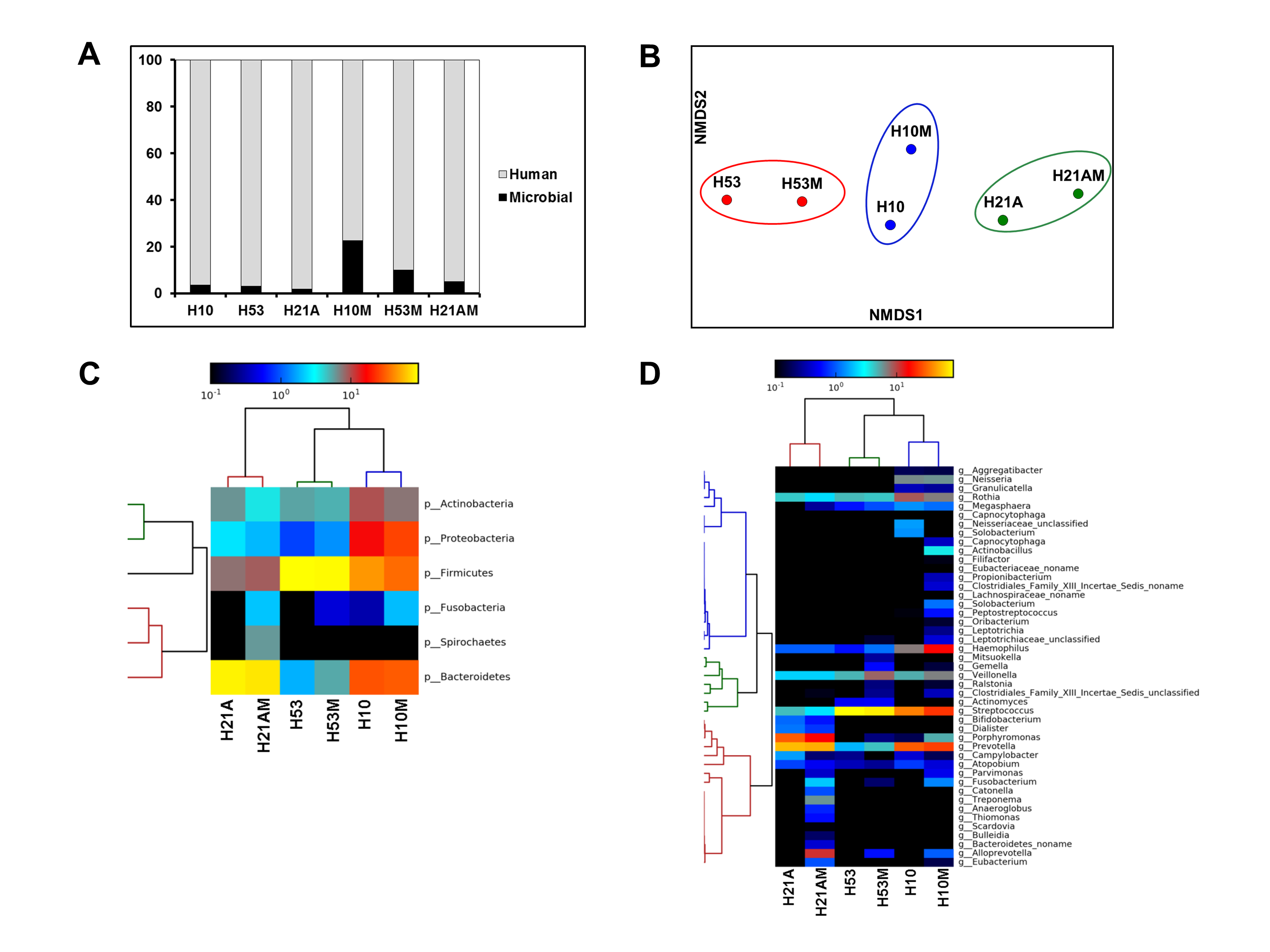

Supplement: Supplementary file 2 — Figure S1. Comparison of the esophageal microbiome prior to and after enrichment for microbial reads. Figure S2. Effects of proton pump inhibitors and gender on esophageal microbiome composition. Figure S3. Esophageal microbial signatures associated proton pump inhibitor use. Figure S4. Effects of proton pump inhibitors and gender on functional pathways within esophageal microbiome. Figure S5. Esophageal microbiome functional signatures associated proton pump inhibitor use. Figure S6. Negative controls relevant to this study. (ZIP 3954 kb) [file 40168_2018_611_MOESM2_ESM.zip › Eso1 Additional figure 1 New.tif]

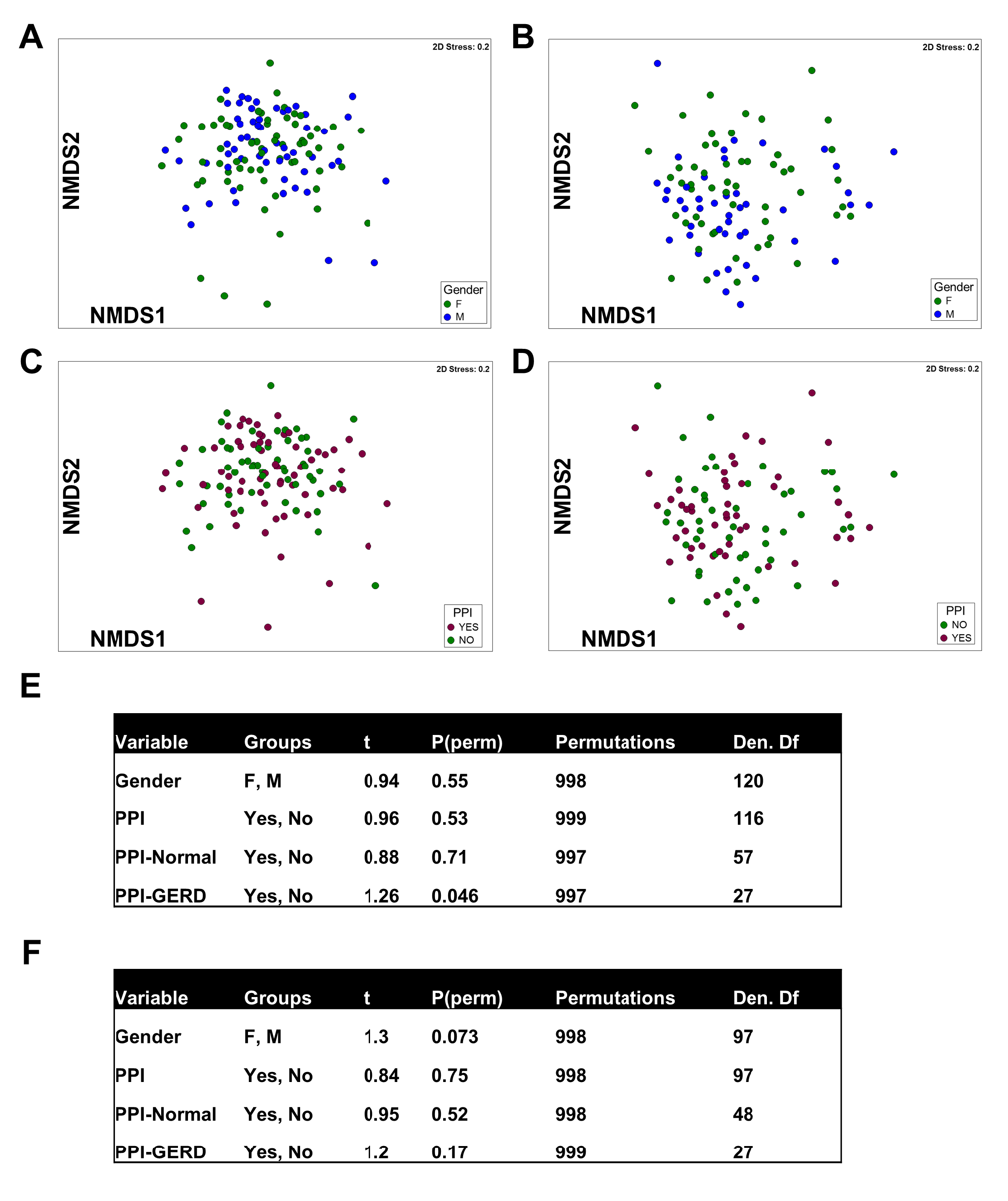

Supplement: Supplementary file 2 — Figure S1. Comparison of the esophageal microbiome prior to and after enrichment for microbial reads. Figure S2. Effects of proton pump inhibitors and gender on esophageal microbiome composition. Figure S3. Esophageal microbial signatures associated proton pump inhibitor use. Figure S4. Effects of proton pump inhibitors and gender on functional pathways within esophageal microbiome. Figure S5. Esophageal microbiome functional signatures associated proton pump inhibitor use. Figure S6. Negative controls relevant to this study. (ZIP 3954 kb) [file 40168_2018_611_MOESM2_ESM.zip › Eso1 Additional figure 2.tif]

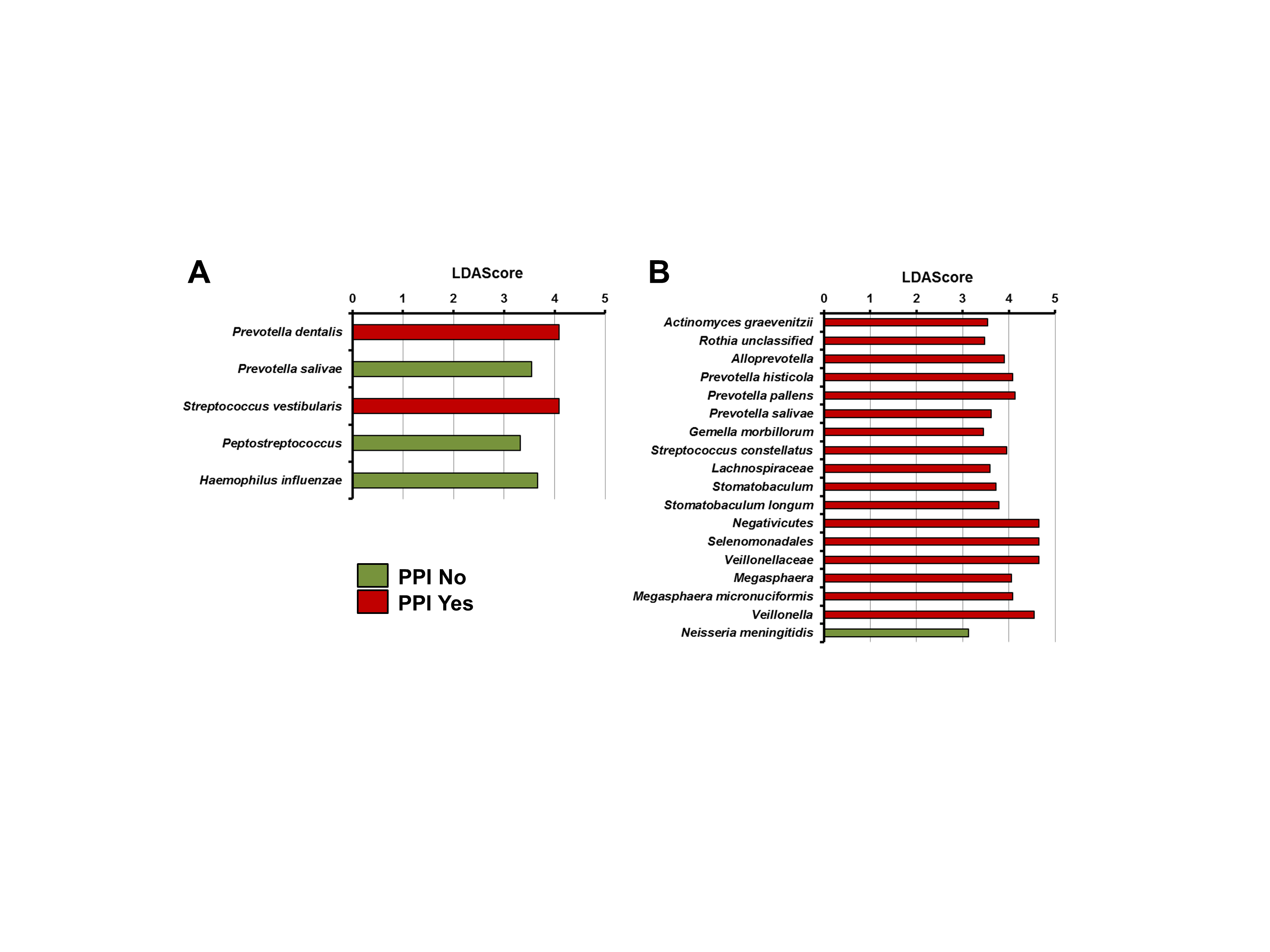

Supplement: Supplementary file 2 — Figure S1. Comparison of the esophageal microbiome prior to and after enrichment for microbial reads. Figure S2. Effects of proton pump inhibitors and gender on esophageal microbiome composition. Figure S3. Esophageal microbial signatures associated proton pump inhibitor use. Figure S4. Effects of proton pump inhibitors and gender on functional pathways within esophageal microbiome. Figure S5. Esophageal microbiome functional signatures associated proton pump inhibitor use. Figure S6. Negative controls relevant to this study. (ZIP 3954 kb) [file 40168_2018_611_MOESM2_ESM.zip › Eso1 Additional figure 3 New.tif]

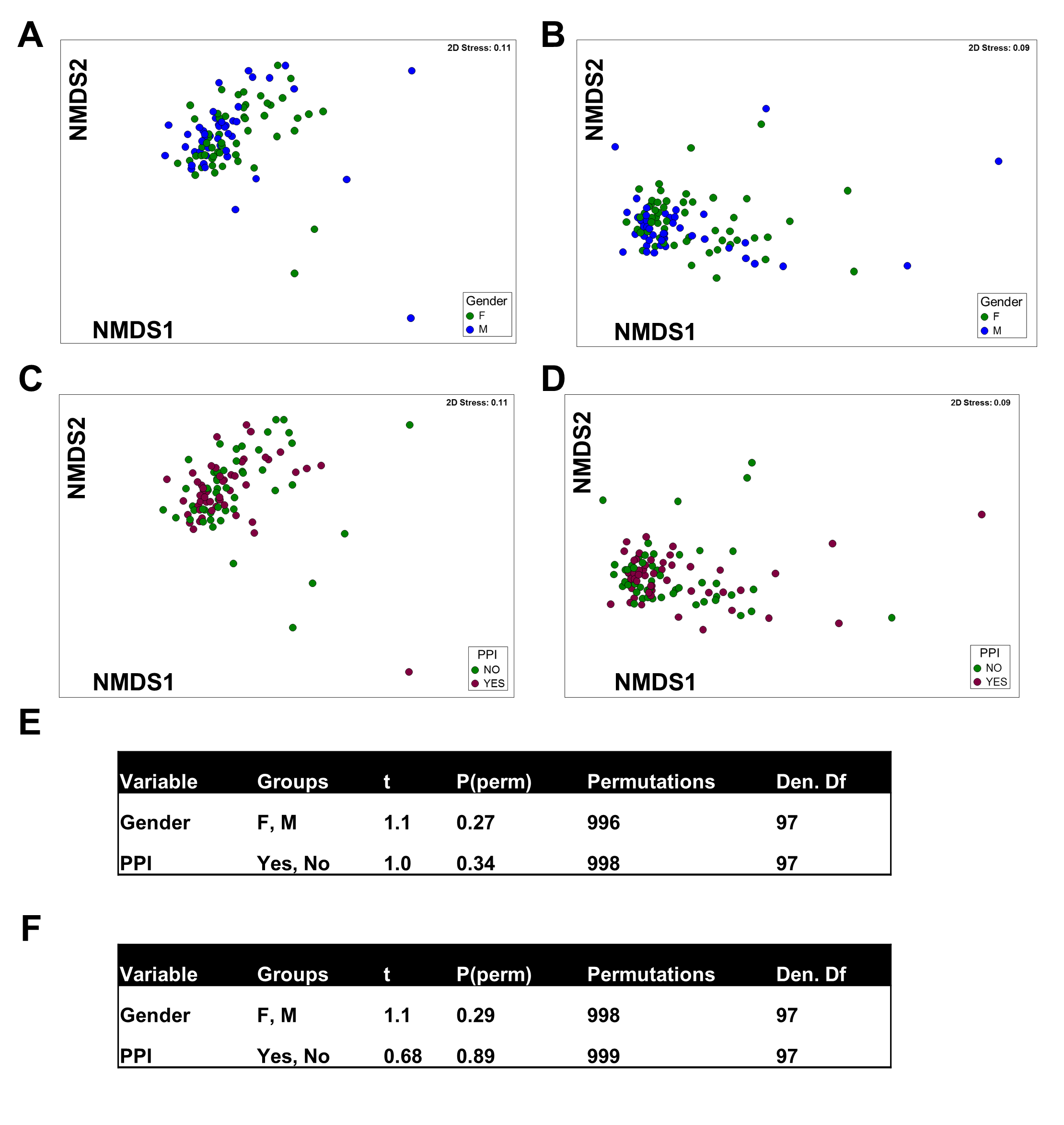

Supplement: Supplementary file 2 — Figure S1. Comparison of the esophageal microbiome prior to and after enrichment for microbial reads. Figure S2. Effects of proton pump inhibitors and gender on esophageal microbiome composition. Figure S3. Esophageal microbial signatures associated proton pump inhibitor use. Figure S4. Effects of proton pump inhibitors and gender on functional pathways within esophageal microbiome. Figure S5. Esophageal microbiome functional signatures associated proton pump inhibitor use. Figure S6. Negative controls relevant to this study. (ZIP 3954 kb) [file 40168_2018_611_MOESM2_ESM.zip › Eso1 Additional figure 4.tif]

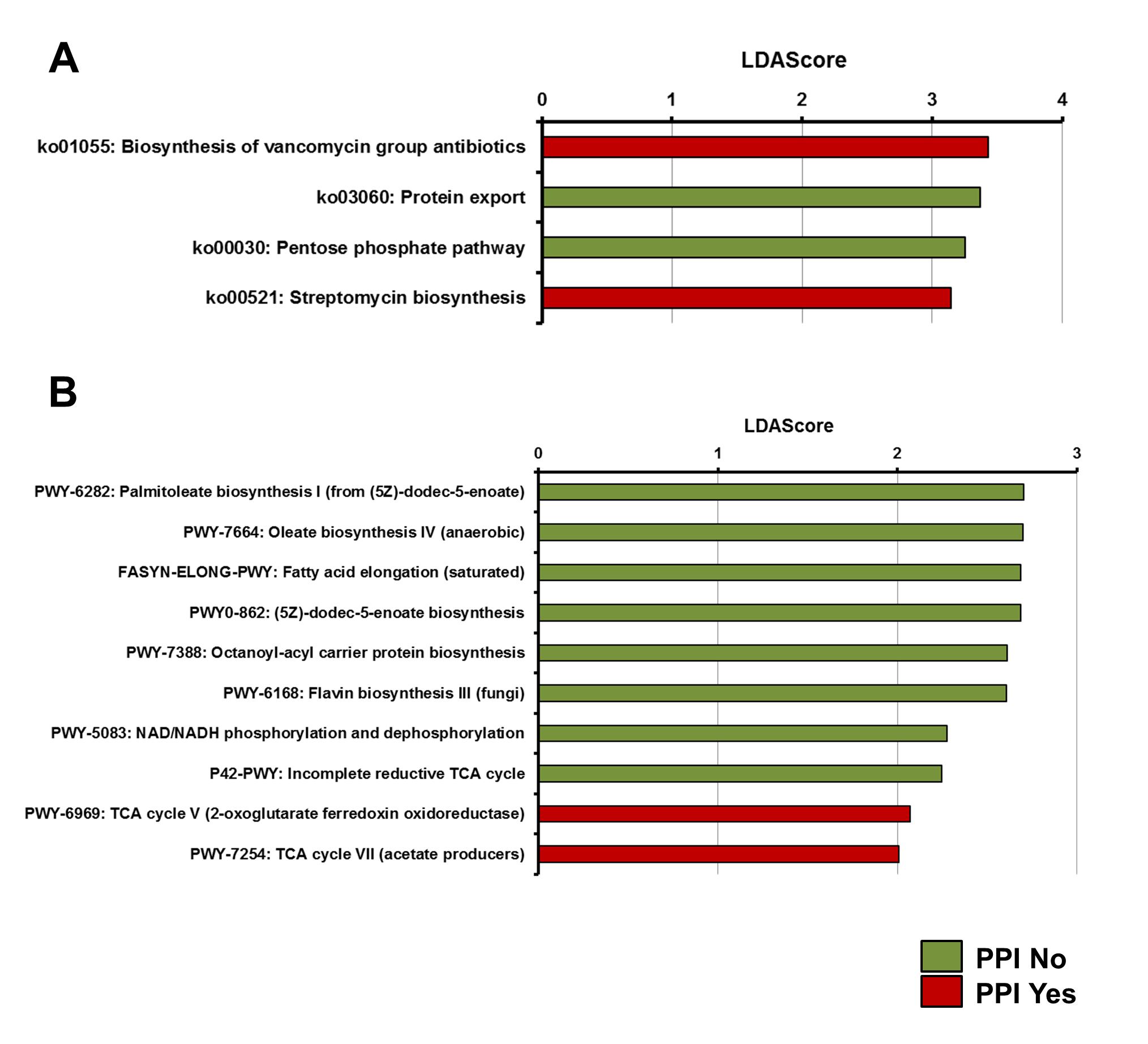

Supplement: Supplementary file 2 — Figure S1. Comparison of the esophageal microbiome prior to and after enrichment for microbial reads. Figure S2. Effects of proton pump inhibitors and gender on esophageal microbiome composition. Figure S3. Esophageal microbial signatures associated proton pump inhibitor use. Figure S4. Effects of proton pump inhibitors and gender on functional pathways within esophageal microbiome. Figure S5. Esophageal microbiome functional signatures associated proton pump inhibitor use. Figure S6. Negative controls relevant to this study. (ZIP 3954 kb) [file 40168_2018_611_MOESM2_ESM.zip › Eso1 Additional figure 5.tif]

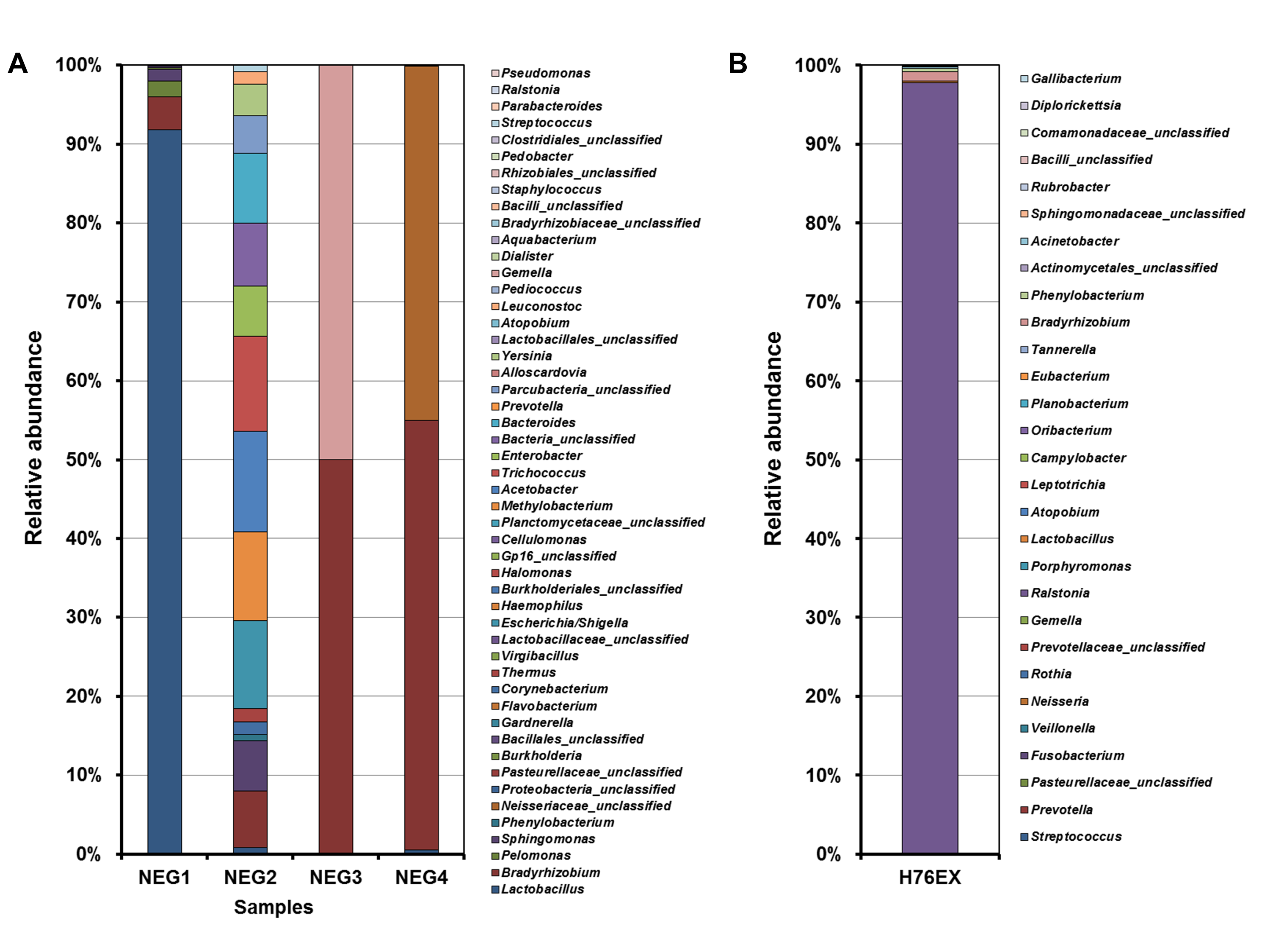

Supplement: Supplementary file 2 — Figure S1. Comparison of the esophageal microbiome prior to and after enrichment for microbial reads. Figure S2. Effects of proton pump inhibitors and gender on esophageal microbiome composition. Figure S3. Esophageal microbial signatures associated proton pump inhibitor use. Figure S4. Effects of proton pump inhibitors and gender on functional pathways within esophageal microbiome. Figure S5. Esophageal microbiome functional signatures associated proton pump inhibitor use. Figure S6. Negative controls relevant to this study. (ZIP 3954 kb) [file 40168_2018_611_MOESM2_ESM.zip › Eso1 Additional Figure 6 New.tif]
